# Randomized double-blind personalized N-of-1 clinical trial to test the safety and potential efficacy of TJ-68 for treating muscle cramps in amyotrophic lateral sclerosis (ALS): study protocol for a TJ-68 trial

**DOI:** 10.1186/s13063-023-07424-8

**Published:** 2023-07-10

**Authors:** Hiroshi Mitsumoto, Ken Cheung, Björn Oskarsson, Howard F. Andrews, Grace E. Jang, Jinsy A. Andrews, Jaimin S. Shah, Joseph Americo Fernandes, Martin McElhiney, Regina M. Santella

**Affiliations:** 1 Department of Neurology, Columbia University Irving Medical Center, 710 West 186 St, New York, NY 10032 USA; 2 Department of Biostatistics, Mailman School of Public Health, Columbia University, 722 W 168Th St, New York, NY 10032 USA; 3 Department of Neurology, Mayo Clinic Jacksonville, 4500 San Pablo Rd S, Jacksonville, FL 32224 USA; 4 Data Coordinating Center (DCC) at New York State Psychiatric Institute and Columbia University, 722 W 168Th St, New York, NY 10032 USA; 5 Department of Neurology, University of Nebraska, 4242 Farnam Street, Suite 650, Omaha, NE 68198 USA; 6 Department of Psychiatry, Columbia University Irving Medical Center and New York State Psychiatric Institute, 722 W 168Th St, New York, NY 10032 USA; 7 Department of Environmental Science, Mailman School of Public Health, Columbia University, 722 W 168Th St, New York, NY 10032 USA

**Keywords:** Kampo, TJ-68, Shakuyakukanzoto, Muscle cramps, ALS, Personalized clinical trial, N-of-1 trials, Randomized controlled study, Muscle cramp scale, Study protocol

## Abstract

**Introduction/aims:**

Muscle cramps are a common and often disabling symptom in amyotrophic lateral sclerosis (ALS), a devastating and incurable neurodegenerative disorder. To date, there are no medications specifically approved for the treatment of muscle cramps. Ameliorating muscle cramps in ALS may improve and sustain quality of life. A widely prescribed traditional Japanese (Kampo) medicine against muscle cramps, shakuyakukanzoto (TJ-68), has been studied in advanced liver disease, spinal stenosis, kidney failure, and diabetic neuropathy. The Japanese ALS Management Guideline mentions TJ-68 for difficult muscle cramps in ALS. Therefore, the rationale of our trial is to investigate the safety and effectiveness of TJ-68 in treating painful and disabling muscle cramps in people with ALS outside of Japan. Accordingly, we are conducting a randomized clinical trial to test the safety and efficacy of TJ-68 in participants with ALS reporting frequent muscle cramps using an innovative, personalized N-of-1 design. If successful, TJ-68 may be used for muscle cramps in a broader population of people with ALS.

**Methods:**

This is a two-site, double-blind, randomized personalized N-of-1 early clinical trial with TJ-68. At least 22 participants with ALS and daily muscle cramps will receive drug or placebo for 2 weeks (one treatment period) followed by a 1-week washout in a four-period cross-over design. While the primary objective is to evaluate the safety of TJ-68, the study has 85% power to detect a one-point shift on the Visual Analog Scale for Muscle Cramps Affecting Overall Daily Activity of the Columbia Muscle Cramp Scale (MCS). Secondary outcomes include the full MCS score, a Cramp Diary, Clinical Global Impression of Changes, Goal Attainment Scale, quality of life scale and ALS functional rating scale-revised (ALSFRS-R).

**Discussion:**

The study is underway. A personalized N-of-1 trial design is an efficient approach to testing medications that alleviate muscle cramps in rare disorders. If TJ-68 proves safe and efficacious then it may be used to treat cramps in ALS, and help to improve and sustain quality of life.

**Trial registration:**

This clinical trial has been registered with ClinicalTrials.gov (NCT04998305), 8/9/2021.

**Supplementary Information:**

The online version contains supplementary material available at 10.1186/s13063-023-07424-8.

## Introduction

### Background and rationale {#6a}

Although a few disease-modifying drugs have been approved for the treatment of amyotrophic lateral sclerosis (ALS) [1–8], these medications only provide modest benefits in slowing disease progression. Therefore, ALS remains a devastating disease with no cure [9]. Not only is ALS almost invariably fatal but many disabling symptoms occur during the course of the disease, one of the most common being muscle cramps. Identifying effective medications that can reduce disabling symptoms could help maintain quality of life longer over the ALS disease course [10]. In fact, there are important examples for significantly improving ALS symptoms: Botox salivary gland injections [11] or radiation [12], dextromethorphan-quinidine for pseudobulbar affect [13], and modafinil for fatigue [14].

Muscle cramps affect 74–95% of patients with ALS [15]. In some patients, muscle cramps are the presenting symptom [16] and may precede weakness [17]. There have been a few clinical trials that have specifically targeted muscle cramps in ALS [18, 19], and several drugs have been offered as treatment for cramps [20, 21]. Recently, mexiletine has shown to reduce muscle cramps in patients with ALS [18]. It was also tested as a disease-modifying medication in ALS. Although the study found no benefits in changing the course of the disease, it was found to ameliorate muscle cramps [22]. However, mexiletine has an FDA black box warning due to potential safety problems relating to risk of fatal cardiac arrhythmias. Thus, there is currently no safe and effective medication for treating muscle cramps for patients with ALS [23].

TJ-68 (manufactured by Tsumura & Co., Tokyo, Japan), known by its generic name Shakuyakukanzoto, is a traditional Japanese medicine (Kampo) and has been widely prescribed in Japan [24, 25] for treating muscle cramps and pains of various causes, including in cirrhosis [26], in lumbosacral stenosis [27], in hemodialysis [28], and in diabetic neuropathy [29].

The drug combination has ancient origins, having been described as a treatment more than 2000 years ago. The modern production of TJ-68 granules uses two medicinal plants, peony root (paeony root), and Glycyrrhiza (licorice root; liquorice root), which contain a multitude of biologically active compounds, and is approved by the Japanese Pharmaceuticals and Medical Devices Agency, with thorough quality manufacturing controls. TJ-68 has been widely used in Japan (e.g., about 2,300,000 estimated annual patients in the year 2019), and the mechanism of antispasmodic and analgesic actions is being studied [30–34]. The Japanese Society of Neurology ALS Treatment Guidelines in 2013 recommended TJ-68 in patients with ALS who suffer from difficult muscle cramps [35]. Surprisingly, this medicine that is commonly prescribed in Japan has never been introduced into a broader population of people with ALS. Therefore, the rationale of our trial is to investigate the safety and effectiveness of TJ-68 in treating painful and disabling muscle cramps in people with ALS outside of Japan. Kampo medicines are used in an individual fashion based on the characteristics of the patient’s constitution and symptoms. In this study, TJ-68 is being evaluated using a personalized N-of-1 trial method.

(Further description of TJ-68 studies done in Japan is available in the Journal Supplement.)

### Explanation for choice of comparators {#6b}

In an N-of-1 trial, outcomes are measured for each patient during alternating, randomly sequenced periods of treatment and non-treatment.

### Specific objectives and hypotheses {#7}

#### Primary and secondary objectives of the present study

**Fig. 1:**
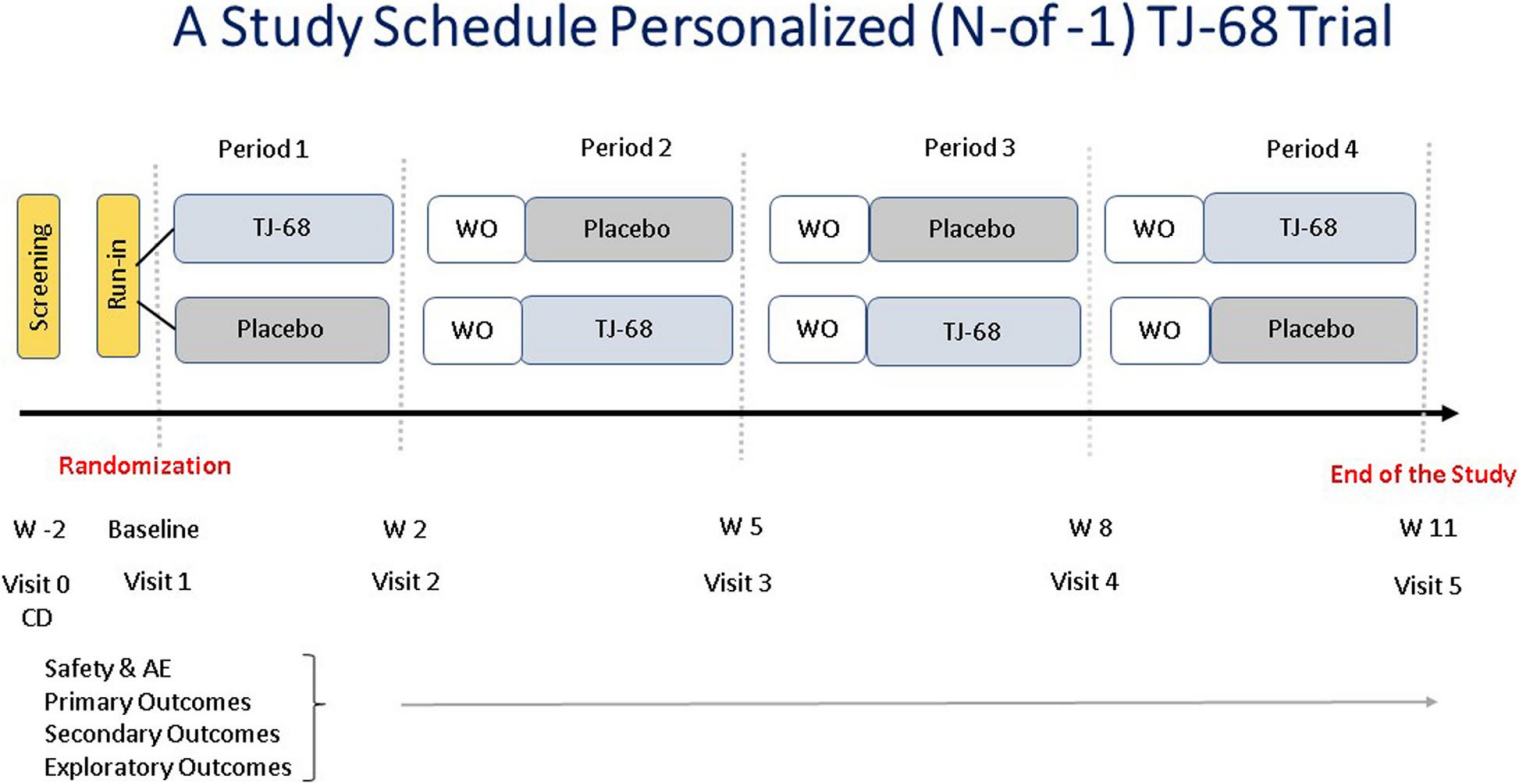
Randomization schedule. We plan to randomize participants in a 1:1 ratio to two treatment sequences: ABBA or BAAB. Each study medication phase is followed by 1 week washout period. WO, washout; W, week; CD, cramp diary; and AE, adverse events

The primary objective of our study is to demonstrate the safety and potential efficacy of TJ-68 for ameliorating muscle cramps in participants with ALS. The secondary objective is to determine if eight secondary endpoints (see below) show positive changes consistent with a benefit of TJ-68 (Fig. 1).

### Trial design

**Fig. 2:**
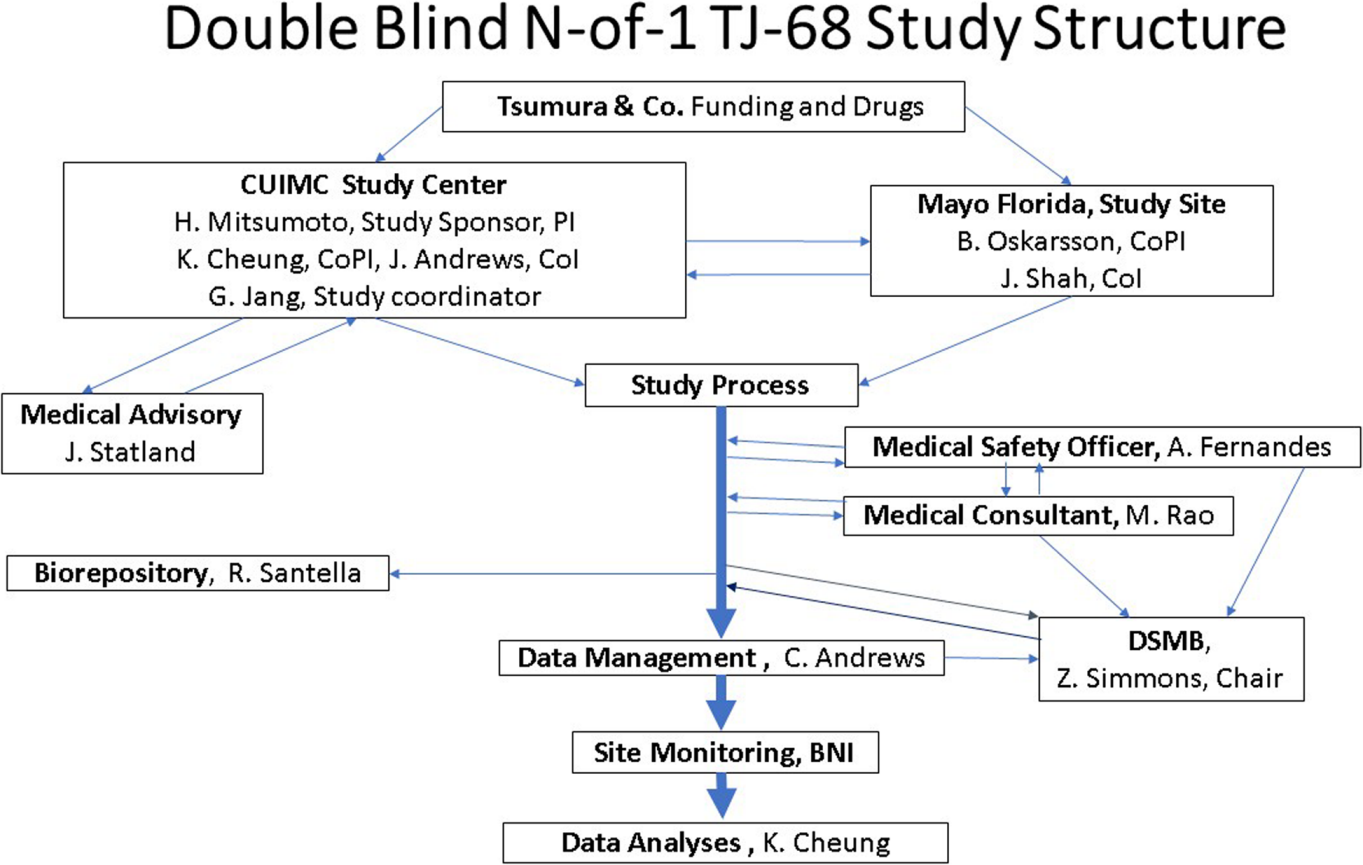
Structure of the study. The research and administrative structure is schematically described. DSMB, data and safety monitoring board; BNI, Barrow Neurological Institute

A two-site, randomized, placebo-controlled double-blind, multi-period crossover (N-of-1) study design. The allocation ratio is 1:1. The framework includes superiority analyses on the primary outcome and secondary outcomes (Fig. 2).

## Methods: participants, interventions, and outcomes

### Study setting {#9}

Academic hospitals specializing in ALS clinical care in USA (New York, New York, and Jacksonville, Florida).

### Inclusion and exclusion criteria {#10}

Table 1 describes the criteria. Participants must have ALS based on the recently accepted diagnostic criteria [36, 37].

**Table 1:** Inclusion and exclusion criteria

**Inclusion criteria**
1. Study participants are included if they meet the following inclusion criteria: 2. Diagnosed with ALS, PMA, or PLS based on the El Escorial ALS Diagnostic Criteria or based on more recently revised Gold Coast ALS diagnostic criteria [35, 36] 3. Experience at least 5 muscle cramp per week 4. Age 20–84 years old 5. Forced vital capacity is 45% of normal or greater in a seated position 6. Able to swallow liquid via the mouth or be given via a feeding tube 7. Caregiver available to assist with speaking or writing on behalf of the participant if they are not able to speak or write due to the disease 8. Understand the informed consent procedure and are able to give informed consent 9. Willing study participants are included if they meet the following inclusion criteria: 10. to commute to the study site for frequent visits, including a screening visit (study visits at the end of weeks 2, 5, 8, and 11) 11. Taking a stable dose of riluzole (Rilutek®), edaravone (Radicava®), AMX0035 (relyvrio ®), or all three both for at least a month before randomization and not expected to require dose titration or initiation of these medications during the study period 12. Willing to discontinue over-the-counter (OTC) products containing peony root, Glycyrrhiza, or both 13. Willing to discontinue mexiletine, quinine sulfate, or ranolazine during the study period 14. Willing to avoid food, beverages, and medications that may induce or inhibit metabolism enzyme or transporters 15. Willing to refrain from initiation or dose adjustment of baclofen, gabapentin, pregabalin, and/or memantine during the study period (stable dosing of these medications is allowed) 16. Willing to practice contraceptive measures for male and female patients
**Exclusion criteria**
1. Study participants will be excluded from the study based on the following exclusion criteria: 2. History of allergic reactions to peony root, Glycyrrhiza, or FD&C Yellow No. 5 (tartrazine) 3. Takes any medication known to increase the risk of pseudoaldosteronism or hypokalemia, including corticosteroids and diuretics (except potassium sparing diuretics, such as spironolactone or amiloride) 4. History of pseudoaldosteronism or hypokalemia or current use of potassium supplementation 5. Screening potassium level 3.4 mEq/L or less 6. Screening diastolic blood pressure (DBP) more than 90 mmHg or systolic blood pressure (SBP) more than 150 mmHg after sufficient rest 7. Screening albumin below normal laboratory level either at the Columbia or Mayo Clinic Jacksonville Laboratory 8. Screening bicarbonate or carbon dioxide level greater than 29 mmol/L, suggesting metabolic alkalosis 9. Screening sodium level greater than 145 mmol/L, suggesting hypernatremia 10. Unstable or active medical or neurological (other than ALS) diseases which require treatment 11. Failure of the Capacity Assessment [38] 12. Not able and/or willing to comprehend and sign the informed consent 13. Not able to speak or write English to complete the primary outcome measure, the MCS 14. Taking any experimental medication or unapproved medications directed at treating muscle cramps 15. Those who are pregnant or breastfeeding 16. Those who have renal or hepatic impairment

### Intervention for each group {#11a}

The planned daily dose of TJ-68 or placebo is 2.5 g t.i.d. For dosing, one sachet of granules will be dissolved in approximately 1 oz of lukewarm water and must be administrated before meals in solution. To prevent potential food-drug interactions, alcohol, caffeine, St. John’s wort, grapefruit, and ginkgo biloba leaf extract should be avoided during the intake of the study drug. The participants attend visits 2, 3, 4, and 5 after each period without taking the study medication (other concomitant medications can be taken). Participants will attend without eating breakfast (unless participants have impending weight loss; the status of breakfast food intake for these participants will be documented). Drinking water will be allowed. The participant takes the first medication dose at the baseline visit while the coordinator watches.

#### Dose rationale

The dosage of 2.5 g three times a day was approved for TJ-68 by the Japanese regulatory authority in 1986. Many patients in Japan receive the approved dosage to treat muscle cramps of various origins (liver cirrhosis, renal failure, diabetic neuropathy, ALS, nocturnal cramps in the elderly, sports-related muscle cramps, etc.).

#### Investigational product

The TJ-68 extract granules and matching placebo are manufactured according to Good Manufacturing Practices and supplied by Tsumura & Co. (Tokyo, Japan). One packet of the study drug is administered orally three times a day, before meals in solution. The intervention group receives TJ-68 extract granules at a daily dose of 7.5 g which contains equal amounts of peony root and Glycyrrhiza. The placebo group receives granules prepared from lactose and other ingredients not containing TJ-68 extract powder, with an appearance and taste formulated to be as similar as possible to those of TJ-68. The matching placebo contains corn starch, lactose hydrate, dextrin, magnesium stearate, FD&C blue no.1 aluminum lake, FD&C yellow no.5 aluminum lake, and red ferric oxide.

There is no need for diet control in this study, but the following two recommendations will be given. Participants should refrain from intake of a large quantity of Glycyrrhiza-containing food, e.g., licorice candy. Food containing high levels of potassium will be recommended to all participants because TJ-68 is known to deplete potassium, which can be associated with adverse events (AEs) or symptoms.

### Criteria for discontinuing or modifying interventions {#11b}

Patients will be informed that they are free to discontinue the study drug or withdraw from the study at any time and for any reason. The investigators may discontinue the study drug or withdraw a patient from the study if they believe it is not in the best interest of the patient to continue the study.

#### Study drug interruption

Participants who do not take the total assigned daily dose of the study drug due to hospitalization or other circumstances will be encouraged to return to treatment. The study PI (HM) and Co-PI (BO) will be contacted if a participant has discontinued treatment for more than 1 week (prolonged study drug interruption). If an interruption of more than 1 week has occurred, the investigator will evaluate the participant in the clinic to ensure that it is safe for the participant to resume drug dosing and will consult with the study PI/Co-PI and Medical Safety Officer (MSO), Dr. J. Americo Fernandes (JAF), University of Nebraska, to determine the appropriate course of action.

Participants should be strongly encouraged to perform the early termination visit as soon as possible following the last dose of study drug. The follow-up visit should be performed 14 days after this last dose. Otherwise, patients will be followed by phone. In addition, they should be encouraged to return all remaining study assessments (with the exception of the 12-lead ECG, clinical laboratory) for the duration of the study following drug discontinuation. These remaining study visits should be performed according to the original study visit windows. Participants, who decide to start or stop riluzole, edaravone, or AMX0035 after screening and before randomization, will no longer be eligible for the study and should not be followed. If a participant decides to start riluzole, edaravone or AMX0035 after randomization, they will be terminated from the study by the site. They should return to the clinic for their early drug termination visit as soon as possible following the last dose of the study drug and return 14 days after the last dose for the follow-up visit. They will not be followed thereafter. For participants who terminate early from the study and do not want to return to the clinic for the visits described above, the evaluators will call the participants to collect the information over the phone if the participant is willing. The PI (HM) and Co-PI (BO) will be immediately notified of any participant discontinuation. In case of withdrawal of study participation, efforts will be made to perform early termination and follow-up visit assessments. The date the patient withdraws from the study and the reason for discontinuation will be recorded on the patient’s electronic clinical report form (eCRF). All patients who prematurely discontinue from the study for AEs will be followed for up to 30 days by visit or phone (until the AE resolves or until the unresolved AE is judged by the Investigator to have stabilized).

### Strategies to improve adherence and procedures for monitoring adherence {#11c}

Telephone interviews are incorporated into the study design to ease burden of participation and are administered routinely, twice a week, to assess the muscle cramp scale (MCS). At the same time, study protocol procedures and study drug dosing are reviewed to encourage adherence to the study medication.

#### Study drug accountability and disposal

Participants are asked to keep a drug diary to record the day and time they took the study medication. Participants are instructed to keep empty drug packets and to bring them to their next site visit. Empty or unused drug packets are kept until the entire study is complete. Study drug dispensation will be tracked by research pharmacy at each institution and study coordinator will collect all used and unused study drug to monitor study drug accountability and monitor participant compliance with dosing. Study drug will be disposed according to each institution’s standard operating procedures for study drug disposal and destruction.

### Relevant concomitant care and interventions that are permitted or prohibited {#11d}

As stated in the inclusion and exclusion criteria, mexiletine, quinine sulfate, and ranolazine are not allowed as concomitant medications due to a potential for confounding the primary outcome. We will not allow the initiation of any medications commonly prescribed for muscle cramps for the first time while participating in the study. These include baclofen, gabapentin, pregabalin, and memantine.

From a safety standpoint the following medications are well known to reduce serum potassium and/or induce pseudoaldosteronism and are therefore contraindicated. They include diuretics (loop diuretics and thiazide diuretics), corticosteroids, thyroid hormone, digoxin, β-blocker, and insulin. Multidrug resistance-associated protein 2 inhibitors are also prohibited because they interfere with excretion of the sulfate conjugate of Glycyrrhiza into the bile duct; this includes probenecid, cyclosporine, and certain antiviral drugs [39, 40].

### Outcomes {#12}

#### Columbia Muscle Cramp Scale (MCS)

Our research team at Columbia University developed the MCS [40] which consists of 5 items, takes only a few minutes to complete and can be accurately and effectively administered in person or by telephone. One major advantage of the MCS over the cramp diary is that it is administered by an evaluator, reducing recall bias and lack of insight, which can limit subjective assessments. Based on the FDA’s recommendation to our Pre-IND inquiry (PIND 152896), we have decided to use #5 item, visual analog scale (VAS) (0–10) for Muscle Cramps Affecting Overall Daily Activity of Columbia Muscle Cramp Scale (MCS) [41] as the primary outcome in this trial.

#### Cramp Diary (CD) and cramp pain scale

CDs have been used in previous clinical trials including cannabis and mexiletine [17, 19, 22]. The CD prompts the recording of daily cramp frequency and severity in an individual’s arms, legs (right and left), and torso. We also separately investigate the degree of cramp pain based on a visual analog scale [41]. We use CD as the secondary outcome in our study and ask participants to make a CD entry twice a week throughout the entire study. However, during the first week after the screening visit and before the baseline visit, participants are required to keep a daily CD for determining study eligibility.

#### ALSFRS-R (ALS Functional Rating Scale-Revised)

ALSFRS-R is the most widely used and also the most widely validated scale against other clinical measures assessing ALS disease progression [42].

#### Clinical Global Impression of Changes (CGIC)

The CGIC score is widely used as a patient reported outcome (PRO) measure of clinically meaningful change, distinct from an instrument’s ability to assess changes in general [43]. This scale is evaluated by the participants themselves and by an independent evaluator (the study coordinator).

#### Goal Attainment Scale (GAS)

In a rare disease such as ALS, it is often the case that study participants are in different stages of the disease, and heterogeneity within the study population compromises treatment evaluation. GAS is an individualized instrument that allows patients to set their own treatment goals with their treating physician and to evaluate the effect of an intervention on an individual basis. GAS is considered an important PRO [43–45].

#### ALSAQ-5 (ALS assessment of quality-5)

ALSAQ-5 (ALS assessment of quality-5) is a simple and short quality of life measure. It is an efficient and valid PRO measure for quality of life [46].

#### Columbia-Suicide Severity Rating Scale

The Columbia-Suicide Severity Rating Scale is an effective, short suicide assessment tool for evaluating suicidal ideation and behavior, which is FDA approved. It will be administered at each in-person study visit [47].

### Time schedule {#13}

**Fig. 3:**
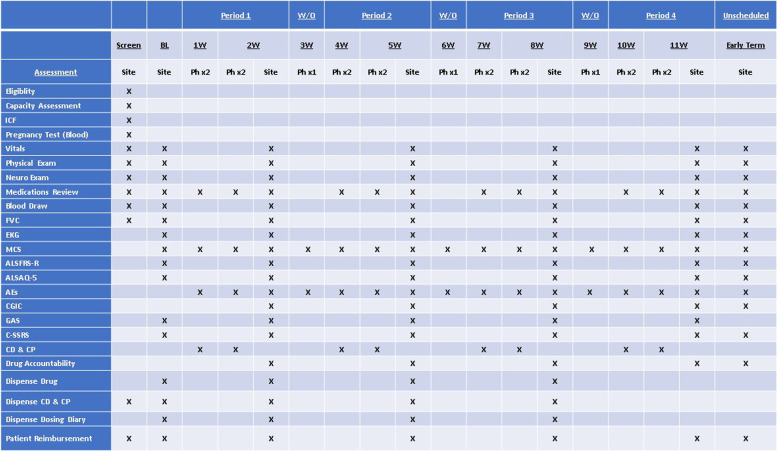
Study schedule. The figure provides details on the study schedule from screening to the end of the study, including baseline, telephone visits, and site visits after each treatment phase. W/O, washout; BL, baseline; W, week; Site, site visit; Ph, phase; ICF, informed consent form; FVC, forced vital capacity; EKG, electrocardiogram; MCS, muscle cramp scale; ALSFRS-R, ALS functional rating scale-revised; ALSAQ-5, ALS assessment of quality-5; AEs, Adverse events; CGIC, clinical global impression of changes; GAS, goal assessment scale; C-SSRS, Columbia-suicide severity rating scale; CD, cramp diary; and CP, cramp pain scale

Figure 3 outlines the study schedule.

#### Screening visit

The investigators or study staff will explain the study in full detail, go over the informed consent, and answer any questions. After a clinical assessment for capacity assessment [38], the participant provides informed consent. Then, study assessments will be completed as specified in Fig. 3. Finally, the study team will review how to complete a daily cramp diary (CD, 42) with each participant.

#### Baseline visit

This visit is scheduled at least 1 week after the screening visit. The participant brings CDs so that the study team can review how many cramps were experienced in the preceding week. At this visit, the study team will confirm that the participant experiences, on average, at least one muscle cramp a day for a week. When this is confirmed, the participant is randomized and assigned a study number. Participants are assigned to one of two sequence groups (see Fig. 2), and the site research pharmacy, based on the randomization assignment, provides the study coordinator with the first packet containing the study medications for the next 2 weeks. Examinations and assessments as shown in Fig. 3 are conducted. Following completion of the visit study procedures, the study coordinator gives the first packet containing a 3-week supply of study medication in case of any delayed appointment to the participant with the assigned study number. The participant takes the first medication dose at the baseline visit while the coordinator watches. Instructions on how to keep empty TJ-68 drug packets along with a dose diary for taking the medication are provided.

#### Weekly telephone calls

Telephone interviews are administered routinely twice a week for the MCS. AEs are assessed and recorded. Any changes in other medications are checked and confirmed. The participants complete a CD and Pain Scale twice a week to record episodes of muscle cramps and pain experienced in the preceding 24 h.

#### End of phase (1, 2, 3, and 4) visits

As shown in Fig. 3, we repeat the procedures of the baseline visit. In addition, AEs are assessed and CGIC is completed by the participant and the evaluator. GAS is assessed based on expectations set at the baseline visit. The participant brings the CD and cramp pain questionnaires to the visit. The dose diary is reviewed, and the number of used and unopened TJ-68 packs are counted and recorded for drug accountability. At the end of the visit, the participant receives the assigned packet containing the study medication for the next 2-week period, which is taken from this day onward to the next period.

#### The washout period

At the end of each of the first three 2-week periods, the participant proceeds to a 7-day washout period (see Figs. 2 and 3). During this 7-day washout period, the participant does not take the study medication. At the end of the 7-day washout period, participants receive a phone visit to check the MCS #5, and any AEs. No site visit is necessary. General instructions for starting period 2 are provided.

The 7-day washout period is justified because the previous pharmacokinetic (PK) study done by the Tsumura research team [33], targeting six active constituents after a single oral dose of 2.5 or 5.0 g/day, showed that the longest *t*_1/2_ was for glycyrrhetic acid, whose *C*_max_ was approximately 200 ng/mL, at approximately 10 h. We assumed at least 5 times the PK half-life would be required for a washout period. For glycyrrhetic acid, it is approximately 50 h; therefore, a 1-week washout period (168 h) between periods 1 and 2, 2 and 3, and 3 and 4 should be sufficient.

#### The end of the study period, week 11 visit

Identical procedures are repeated at the end of study period 4 (see Fig. 3).

### Sample size {#14}

The sample size was determined based on the Visual Analog Scale endpoint under a four-period crossover design. Specifically, assuming a within-subject standard deviation of the scale at 1.4 based on our previous publication [41], the study was sized to achieve 85% power to detect a one-point shift on the scale at 5% significance (two-sided) and obtained a total sample size of 22. Participants will be randomized in a 1:1 ratio to two possible treatment sequences: ABBA or BAAB. Assuming 15% attrition, we plan to enroll a total of 26 participants, with 13 participants in each of the two treatments.

### Recruitment {#15}

This study is posted on the websites of Northeastern ALS (NEALS) and the ALS Association. The PI and Co-PI provide open webinars on the study when the opportunity arises. IRB-approved study flyers are given to all ALS Clinic personnel, particularly nurse coordinators and study coordinators, to hand to potential participants. The study details are discussed with the faculty of the neurology departments who are specializing in ALS at Columbia and Mayo in an effort to capture all potential candidates. When study staff see new patients with difficult cramps, we also introduce the study with the flyers. These efforts are continuing at both study sites.

We anticipate completing the study recruitment within 2 years from the commencement of the study. If this timeline cannot be met, we will extend the study period with IRB approval.

## Methods: assignment of interventions

### Allocation {#16a}

Treatment sequence was randomized by computer within small blocks, and stratified by Dr. Howard Andrews (HA), director of the Data Coordinating Center at Columbia University, who serves as the unblinded biostatistician. To reduce predictability of a random sequence within blocks, block size was also randomized. The 22 participants will be randomized 1:1 to the two treatment sequences as shown in study schedule (Fig. 3). To ensure power with anticipated dropout (15%), we anticipate randomizing up to 2 additional participants to each treatment sequence.

### Allocation concealment mechanism {#16b}

The allocation sequence is provided to the research pharmacies at both study sites via secured email by HA to prepare and dispense study treatment. All other study personnel involved in direct study conduct and participants are completely blinded to the randomization scheme.

### Implementation {#16c}

HM and his research coordinator at Columbia and BO and his research coordinator at Mayo enroll each study participant and assign the next available subject ID. The research pharmacist assigns an intervention to participants according to the subject ID and study assignment schedule provided by HA.

### Blinding {#17a}

Please see 16a (above). All the investigators, MSO (JAF), a medical consultant (Dr. Rao), and study staff are blinded for the duration of the trial. Authorized research workers at each site are assigned user-specific passwords to enter and edit data from their site but are not able to see or modify data from the other site. The online data are maintained and backed up daily on an institutional REDCap installation, housed in a physically secure server area to which only institutional Internet staff have access. Data in the online system are de-identified: subjects are identified with a consecutive Study ID; names and contact information linked to the Study ID are maintained separately and securely at each study site. The blinded MSO has read-only access to the database so that AEs and other safety data can be monitored continuously. The DCC generates unblinded reports to the data and safety monitoring board (DSMB) with statistical summaries of study performance, medication compliance, and treatment compliance.

### Unblinding {#17b}

A joint agreement between PI and Co-PI will allow unblinding of individual participants on a need-to-know basis and, if necessary, termination of treatment in the event the MSO and investigator determine the participant has had a serious adverse reaction to the study medication. Unblinding of the project statistician who will analyze the data (Ken Cheung) will occur at the end of the study, after the last participant has been evaluated, all data have been entered and cleaned, and the database has been locked.

## Methods: data collection, management, and analysis

### Data collection methods {#18a}

All data at in-person visits and telephone interviews, outlined in Fig. 3, are collected, and entered in the data management system. CRFs are available at the end of the study protocol version 7. All the assessment techniques for outcome measures and other relevant clinical information were reviewed at a kick-off meeting. Following this meeting, ad-hoc virtual training was carried out for the two study sites by the Columbia study coordinator, PI, Co-PI, clinical investigators, and Dr. McElhiney. When coordinators are changed, new training is repeated. Coordinators who assess ALSFRS-R and FVC must receive NEALS Certificates for ALS Clinical Trials.

### Promote participants retention and complete follow-up {#18b}

Every time participants are seen at each site visit and telephone interview, the study protocol and procedures are reviewed and participants are encouraged to stay in the study.

If participants decide to terminate early from the study or when participants are withdrawn for any reason, they will be asked to complete an early termination evaluation. They should be seen in the clinic as soon as possible following discontinuation of the study drug. The following procedures will be performed: vitals, physical and neurological examinations, medication review, AE review, routine safety blood tests, FVC testing, and ECG. The MCS, ALSFRS-R, ALSAQ-5, CGIC, CD, cramp pain questionnaire, and dosing diary will be collected. The patient will return all used and unopen study drug packets (see Fig. 3).

Visit windows: We will make every effort to maintain the scheduled plan and site visits. Due to various unforeseen circumstances, the visit date may need to be changed. We will allow a window that includes 2 days before or 4 days after the anticipated visit date per study protocol. Further deviations will be handled on a case-by-case basis to maximize successful participation without compromising participant safety and ensuring data reliability.

### Data management {#19}

All trial data are obtained by authorized research staff at both study sites, and managed by the CUIMC’s Data Coordinating Center (DCC) (Fig. 3), which has extensive experience in the management and reporting of clinical trial data. The data is managed in a secure online data management system using REDCap functionality, specifically designed for clinical trials. The 4-period crossover design required for this study is mirrored in the REDCap database structure, with REDCap events specified for each of the 4 periods; adverse events (AEs) and other safety-related data are databased using standard structured reporting as “anytime” events that are entered into the system immediately upon occurrence. The data system captures each relevant study domain on a separate structured online form.

### Statistical methods {#20a}

Endpoints of the study. (1) Primary safety endpoint: no clinically significant signs and symptoms, no laboratory findings of hypokalemia, nor other AEs. (2) Primary efficacy endpoint: response to MCS item #5 [41]: “Muscle Cramps Affecting Overall Daily Activity.” We assess the MCS VAS obtained during the second week of each treatment period for comparison with the MCS obtained during the second week of each placebo periods. The study is powered to detect a one-point difference, on average, between the treatment period and the placebo period. (3) There are several secondary endpoints.

#### Planned analyses

The analysis of the primary endpoint is performed by Dr. Ken Cheung, Columbia University, who designed the N-of-1 study and has extensive experience in the analysis of clinical trials data. For the primary outcome, the data will be analyzed using linear mixed models to estimate the average effect of TJ-68 on MCS item #5 when compared to placebo. Specifically, for each participant, VAS assessments in the second week of each of the 2-week periods are averaged, and the average is used as the dependent variable in the mixed models with a random participant effect; data in the first week of each period will not be used to mitigate any potential carryover effects from the treatments in the previous periods. Secondary endpoints will also be assessed in the framework of generalized linear mixed models. Balanced randomization between the two treatment sequences eliminates bias due to a time trend. In the case of a slight imbalance, we will conduct sensitivity analyses by including time period as an independent variable in the mixed models. We will also estimate carryover effects of TJ-68 by estimating the appropriate contrasts in the linear mixed models.

### Any additional analyses {#20b}

We will perform exploratory analysis to understand the treatment mechanism via heterogeneity of treatment effects. Specifically, with multiple observations per treatment per participant, we will use empirical Bayesian hierarchical models [48] to estimate the individual treatment effects and associate these individual effects with baseline variables. This potentially provides information about participant selection in future trials.

### Non-adherence and handling of missing data {#20c}

Every effort will be made to retain each participant until trial completion and to collect complete information on each subject. All efficacy analyses are performed with the modified intent-to-treat (mITT) population sample, as the primary analyses. The mITT population sample consists of all randomized participants with outcomes evaluated in at least two post-randomization periods (with one period in TJ-68 and one in placebo). Note that we replace participants who fail to complete all four treatment periods; therefore, the mITT population may include more than 22 participants.

As sensitivity analyses, we will also perform efficacy analyses with completers only, that is, a participant with outcomes evaluated in all four treatment periods.

Safety analyses are performed as-treated. All AEs and SAEs are recorded by treatment period for each participant and analyzed using mixed logistic regression.

Missing data patterns are compared by treatment sequence as well as treatment periods. While linear mixed effect models are valid under missingness at random, sensitivity analyses are performed using various imputation approaches:

(A) Mean imputation: Missing data within a treatment period are imputed using averages from other data points within the same treatment period. If data in the entire treatment period are missing, the observation from the previous period (i.e., of a different treatment) is carried forward, as a conservative approach.
(B) Worst-outcome imputation: All missing data are imputed using the worst values observed in a treatment group.

## Methods: monitoring

### Data monitoring {#21a}

An independent DSMB was formed to monitor the safety of the study medication and data integrity. Dr. Zachary Simmons, Department of Neurology, Penn State University, is the chair, and the members are listed in Study Group (Fig. 3). Quarterly open DSMB conference calls also include JAF, the blinded MSO, Dr. Maya Rao (a blinded nephrologist/consultant for hypokalemia, CUIMC), Ken Cheung (KC) (blinded biostatistician), the PI, Co-PI, and the Tsumura research team. In closed sessions, where unblinded data are reviewed, Dr. Andrews, the only unblinded member of the study team, will participate as a non-voting member. The DSMB serves as an important independent body to advise the PI on decisions regarding the removal of study participants and data integrity and has the authority to recommend termination of the study by the sponsor based on performance or safety issues. The DSMB provides input to the Sponsor (HM) with a written report/summary of the discussions, as well as any findings and conclusions of each quarterly meeting. In general, an emergency DSMB meeting is prompted by the request of the investigators (HM and BO) when safety concerns are raised. The chair of the DSMB can also call an emergency meeting based on the unblinded data provided by the Data Management Center.

#### Excessive rates of AEs and emergency DSMB meeting

In general, an emergency DSMB meeting will be prompted by the request of the investigators (Drs. Mitsumoto and Oskarsson) when safety concerns are raised. The chair of the BSMD can also call an emergency meeting based on the unblinded data provided by the unblinded statistician, Dr. Andrews.

In a previous controlled clinical trial of TJ-68 among participants with liver cirrhosis, 9 participants reported a treatment-related AE among a total of 90 participants (7 out of 49 with TJ-68 and 2 out 41 with placebo) [25]. Based on the experience of this clinical trial, we will ask to hold an emergency DSMB meeting if a total of 9 phase-specific AEs occur (approximately 10% of 88 total phases in this trial—22 subjectsX4 phases = 88). Furthermore, if two participants report any SAE or deaths are observed during any point in the trial, an emergency DSMB meeting will be immediately initiated.

### Interim analyses and stopping guidelines {#21b}

There are no plans for interim analyses because the study is an early clinical trial and enrolls a small number of participants. The study may be discontinued at the recommendation of the DSMB due to safety reasons such as are excessive rates of AEs, or if there are medical reasons affecting the continued performance of the trial phase, or difficulties in the recruitment of participants, or a decision to cease or delay further development of the drug.

### Harms {#22}

#### Safety issues

Hypokalemia is the main AE of concern for those who take TJ-68. When hypokalemia is mild to moderate, participants who take TJ-68 may develop mild asthenia, fatigue, weakness, edema, muscle cramps, muscle aches, stiffness or spasms, digestive problems, heart palpitations or arrhythmia, tingling and numbness, and breathing difficulties. In severe hypokalemia, participants may experience acute rhabdomyolysis, characterized by severe muscle pain, pigmenturia, high creatine kinase levels, and potential kidney shutdown. TJ-68 may also cause other symptoms, such as hepatic dysfunction and jaundice, rash, redness, pruritus, nausea, vomiting, and diarrhea. Previous experience in Japanese patients indicates the frequency of hypokalemia is generally low.

The clinical investigators (HM and JO) and the MSO (JAF) will closely monitor participants for any early signs of hypokalemia and any other adverse events (Fig. 3). Dosing of individual participants will be stopped and not resumed if treatment-related AEs, changes in vital signs, electrocardiograms, or clinical laboratory results are observed and these changes pose a significant health risk (in the opinion of either the Investigator or the MSO). A blood sample for safety analysis should be collected at the time of the event, as deemed required, or as close as possible to the time of the event. In the event of confirmed, marked hypokalemia or any other laboratory abnormality, it is the investigator’s responsibility to ensure contact with the MSO and Dr. Mitsumoto immediately (i.e., within 24 h of awareness or at the earliest possible time point). Participants with AEs of hypokalemia origin accompanied by plasma potassium abnormalities will be carefully monitored. Each Severe AE (SAE) will be reported to the IRB.

#### Safety management

The clinical investigators (HM and BO) and the MSO (JAM) closely monitor participants for any early signs of hypokalemia. Enrolled participants are instructed to check for swelling of ankles or lower legs and to assess body weight every day. Because patients with ALS often lose weight during the course of the disease, any weight gain may indicate impending edema. We will prepare a sheet for participants with appropriate instructions regarding these potential issues. Increasing BP is another important indicator for potential hypokalemia. BP is determined at every visit while at rest for at least 5 min in a seated position. BP is measured twice and the lower of the two measurements recorded. If BP elevation (either diastolic or systolic) of more than 10 mmHg from the baseline BP is confirmed, we will repeat BP measurement within 1 week. Continued BP elevation is considered significant and may be a possible indicator of potential hypokalemia. Blood potassium levels will be checked at every visit. However, we will decide to obtain ad hoc, at-home blood testing, as deemed necessary if impending hypokalemia is suspected.

The CU Study Center may request the Co-PI (BO) to perform or arrange for the conduct of supplemental measurements and/or evaluations to elucidate as fully as possible the nature and/or causality of any AE/SAE.

The DSMB will periodically assess participant safety in an unblinded manner during the course of the study. No unblinded data will be accessible to the investigators, the study biostatistician, the site staff, or the sponsor before the database is locked. The specific activities and responsibilities of the DSMB are defined in the DSMB Charter for TJ-68 Study and fully described above.

JAF will function as the blinded-MSO. A designated neurologist will be assigned to cover in his absence, if needed. JAF will work closely with Dr. Maya Rao, a clinical nephrologist, CUIMC, who is experienced in treating hypokalemia and pseudoaldosteronism (Fig. 3).

Dosing of individual participants will be stopped and not resumed if probable treatment-related AEs, including changes in vital signs, electrocardiograms, or clinical laboratory results are observed, and these changes pose a significant health risk (in the opinion of either the Investigators or the MSO). A blood sample for safety analysis should be collected at the time of the event, as deemed required, or as close as possible to the time of the event. In the event of confirmed, marked hypokalemia or any other laboratory abnormality, it is the investigator’s responsibility to ensure contact with the MSO and the sponsor/Dr. Mitsumoto immediately (i.e., within 24 h of awareness or at the earliest possible time point). Participants with AEs of hypokalemia origin accompanied by plasma potassium abnormalities should be carefully monitored. Severe AE (SAE) will require a report to the IRB.

### Other safety issues

#### Assessment of causality

The investigator will assign probable causality to each AE and SAE (related or unrelated). When assessing the relationship to the study drug, the investigator will consider the following factors listed in Table 2.

**Table 2:** Consideration of causality of adverse events and expectedness in the course of ALS

**Assessment of causality**	
• Temporal association between the administration of the investigational product and the event • Cessation of the AE following discontinuation of dosing • Recurrence of the AE with reintroduction of study drug, if performed • Similarity to known class effects • Alternative causes, such as known effects of concomitant medications − Pre-existing risk factors − Concurrent illnesses	
**Assessment of expectedness: based on Medical Dictionary for Regulatory Activities (MedDRA)**	
Dysarthria Dysphagia Dyspnea Gait disturbance Involuntary muscle contractions Muscle spasms	Muscle spasticity Muscle weakness Muscle stiffness Pneumonia aspiration Respiratory failure Weight loss

#### Assessment of expectedness

ALS is a progressive and uniformly fatal neurodegenerative disorder associated with relentlessly progressive loss of motor function, including appendicular, craniobulbar, and respiratory function due to the degeneration of the upper and lower motor neurons that control and innervate the voluntary skeletal muscles. In addition to death due to ALS progression, some medical events are anticipated to occur in the study population as signs/symptoms of ALS progression, which may or may not lead to hospitalization (Table 2) (https://www.ich.org/page/meddra). 

#### Follow-up of AEs and SAEs

All SAEs will be followed until resolution, the condition stabilizes, or until the participant is lost to follow-up. Once the seriousness criterion no longer applies to the SAE (e.g., participant is discharged), the corresponding AE eCRF page should be updated. All relevant additional information collected regarding an SAE, including laboratory test reports, consultation reports from other health care professionals, discharge summaries, or other information will be transmitted to CU Study Center with the follow-up SAE report form within 24 h of receipt or awareness.

After the initial recording of an AE/SAE, the investigators will proactively follow the participant. Non-serious AEs that are still ongoing at the end of the study will be reviewed by the investigator to determine if further follow-up is required. The investigator will document on the AE eCRF any/all ongoing non-serious AEs following study termination. If in doubt, the investigator should consult the medical monitor.

If a participant dies during the study or during the follow-up period, Dr. Oskarsson, Co-PI will provide the CU Study Center with a copy of any post-mortem findings, including an autopsy report if obtainable.

Collection and evaluation of AE/SAE and reporting is described in the Online Supplement.

### Auditing {#23}

Clinical site monitoring is conducted to ensure that the rights and well-being of trial participants are protected; that the reported trial data are accurate, complete, and verifiable; and that the conduct of the trial complies with the currently approved protocol/amendment(s), with International Conference on Harmonization Good Clinical Practice, and with applicable regulatory requirement(s). Barrow Neurological Institute (Jeremy Sheffner, MD, Director) conducts the monitoring (Fig. 3).

## Ethics and dissemination

### Research ethics approval {#24}

IRB approvals of the study protocol were obtained from the Institutional Review Boards of Columbia University Irving Medical Center (CUIMC—IRB #AAAT0610) and the Mayo Clinic Florida (21–008166).

### Protocol amendments {#25}

Currently, there are no plans for protocol modifications (protocol version 7).

### Obtaining informed consent (IC) {#26a}

Site PI (HM and BO) and their authorized study coordinators obtain IC.

### Additional consent {#26b}

Authorized study coordinators and their designates obtain blood specimens.

### Confidentiality {#27}

All study personnel at both sites complete Good Clinical Practice (GCP) training and institutionally required human subjects research training. They maintain full confidentiality of research participants.

### Declaration of interest {#28}

PI (HM) and co-PI (BO) and all other participating professionals have given full COI disclosures.

### Access to data {#29}

Biological data and/or specimen obtained in the present research may be used in future research or provided to other researchers or institutions for purposes beyond those stated in the original protocol. Secondary use is only permissible with anonymized data and/or specimens and in a manner that will protect participants’ identities.

Although secondary use is not necessarily anticipated nor specified at the time of participant consent or data/specimen collection, participants will be consented or re-consented as required by subject ethics boards and the applicable regulations.

### Ancillary and post-trial care {#30}

Participants will be contacted with two weeks to assess health status after the completion of the study. A post-study AE/SAE is defined as any event that occurs outside of the AE detection period (after the final follow-up date). Investigators are not obligated to actively monitor AEs of former study participants. However, if the investigator learns of an SAE that he/she considers reasonably related to the investigational product at any time after a participant has been terminated from the study, the investigator will promptly notify the CU Study Center.

Pregnancy is not an AE; however, information on pregnant, female participants and partners of male participants will be collected if the pregnancy occurs after the participant receives the first dose of the investigational product until 10 weeks after the last dose. The pregnancy information and its outcome will be collected using the Pregnancy Report Form. If the pregnancy occurs in a partner of a male participant, the partner’s consent will be obtained before collecting information regarding the pregnancy and its outcome. Any female participant who becomes pregnant during the study is not eligible to continue the study and should complete the study procedures as soon as possible. If a partner of a male patient becomes pregnant during the study, the male participant may opt to continue his participation but must use a barrier method (condom) to prevent fetal exposure.

The Office for Human Research Protections (OHRP) considers unanticipated problems involving risks to participants or others to include, in general, any incident, experience, or outcome that meets all of the criteria summarized in Supplement Table 3.

### Dissemination policy {#31a}

Study results will be communicated to participants, healthcare professionals, and the public via reporting the results at medical society meetings. Most importantly, we plan to publish the study results in peer-reviewed journals.

### Authorship {#31b}

Authorship of the current manuscript and future manuscripts reporting the results of this trial is based on effort critical for developing the trial design and conducting the study. No professional writers are involved with the manuscript. HM (the lead author, PI) and KC (the second author and the lead biostatistician) conceived the study and received a grant from Tsumura & Co. They, along with BO (Co-PI), led the proposal and protocol development. HA developed the data management plan for the study; the plan was implemented and maintained by DM. MM led the outcome management development. GEJ, JAA, JSS, JAF and RS participated in the data collection. HM prepared the first draft, and all authors participated in the draft development and read the final manuscript and approved.

### Public access to the full protocol {#31c}

The dataset is not associated with this study protocol paper. However, the protocol will be made available to the research community after publication. The main clinical trial has just started and is ongoing. At the completion of the trial study, de-identified data will be made available to the research community.

### Informed consent (IC) {#32}

Attached.

### Biological specimens {#33}

Blood samples are processed into a white blood cell aliquot and multiple plasma aliquots and stored in – 80 °C in the Department of Environmental Health Sciences Biomarkers Laboratory for future studies specifically metabolomic analysis (Director: Regina Santella). Metabolomic analyses of participants’ plasma samples are planned in the future. Any further analyses may be undertaken for assisting and developing the future clinical trials with TJ-68.

## Discussion

Personalized N-of-1 clinical trials have emerged as an important trial design in recent years, particularly for rapid development of treatments for a variety of genetic diseases [49]. More recently, however, N-of-1 trials are the design that has been used to identify the optimal personalized treatment for single participants in situations involving evidence for heterogeneity of treatment effects or the lack of a cure [50–52]. The N-of-1 study design can be effectively applied when one can satisfy the following conditions [53]: The disease must be chronic and have considerable clinical heterogeneity and uncertainty. It has frequent recurrent symptoms or recordable events. The medication being tested should have rapid onset of effects and minimal carry over. Although ALS is a progressive disease, it is sufficiently stable when evaluating an investigational drug during an 11-week period. Further, ALS is heterogenous with varied phenotypic expression and uncertain biological mechanisms [9, 54]. Muscle cramps in ALS, although they vary in frequency, are recurrent and frequent symptoms. TJ-68 has a short peak (~ 3 h) and reaches maximum plasma levels quickly with a brief half-life and reportedly a short time to effect. Muscle cramps in ALS are therefore well suited for applying a personalized N-of-1 study design.

The N-of-1 design allows identification of statistical differences during a short period of time with a small study sample, taking into consideration the high between-subject variability of muscle cramps. Potential benefits include the fact that all participants receive the study drug, which is a frequent request from people living with ALS. A multiple-period crossover design provides greater precision than a two-period crossover design in assessing individual treatment effects [55]. This study aims to analyze the data to inform a potential enrichment strategy for further clinical investigations. In other words, to the study may identify specific ALS disease and/or ALS participant characteristics, or specific metabolomic characteristics that can serve as a marker of “responder group.” If successful, the number of required participants in future studies can be reduced. A multiple crossover design has not previously been used in clinical trials focused on ALS; so, in addition to testing the safety and efficacy of TJ-68, this trial will establish the practical feasibility and utility of the multiple-period crossover design in ALS research [51, 52].

Medicines in Western countries are primarily focused on single bioactive chemicals, whereas Japanese Kampo medicines are mostly plant-based substances containing multiple bioactive chemicals. A combination of these chemicals is believed to exert complex biological actions on the human body for managing a symptom that is complex. Recent pharmacokinetic studies indeed show the presence of multiple biochemical components of TJ-68, as described fully in this Journal Supplement. Future metabolomic studies should provide valuable information regarding changes of various biochemicals with and without TJ-68 during the study period.

The efficacy of TJ-68 was demonstrated in clinical trials targeting painful muscle cramps in patients with cirrhosis, dialysis, diabetic neuropathy, and spinal cord disease [26–29]. In a randomized, double-blind placebo-controlled parallel study of muscle cramps in patients with liver cirrhosis, a total of 101 patients were randomly assigned to receive TJ-68 (7.5 g/day) or placebo for 2 weeks. The improvement (decline) in muscle cramp frequency was significantly greater in the TJ-68 group compared to the placebo group [26]. The pharmacodynamic studies of TJ-68 in animal models showed antispasmodic and antinociceptive effects [30, 31]. The constituents of Glycyrrhiza, glycyrrhizin, flavonoids, and/or their metabolites inhibited intracellular calcium influx and abnormal release of calcium from the sarcoplasmic reticulum, leading to inhibition of tetanic contractions. The constituents of peony root, paeoniflorin and albiflorin, affected noradrenergic nervous systems in the descending pain inhibitory pathway in the spinal cord, leading to analgesia. Clearly, more research is needed to understand neurophysiological substrates and mechanisms of TJ-68 activity.

In summary, we are employing a novel trial design in ALS by using the N-of-1 approach. This study will provide information to establish the safety and potential efficacy of TJ-68 in a broader population of people with ALS beyond Japan to manage disabling muscle cramps and potentially improve and sustain quality of life.

## Trial status

The protocol version number: Latest Version: Version 7 and approval date: 8/17/2022.

Recruitment began in June 2022. To date 4 participants have been enrolled.

We anticipate recruitment will be completed in the next 2 years.

## Supplementary Information

**Additional file 1: Supplement Table 1.** Serious Adverse Events (SAEs). **Supplement Table 2.** Consideration of Causality of Adverse Events and Expectedness in the Couse of ALS. **Supplement Table 3.** Dealing with Unanticipated Problems (UPs). **Supplement Table 4.** Removal of Patients from Study Participation. **Supplement Table 5.** Participant’s Withdrawal.

## Abbreviations

AE(s): Adverse event(s)
IND: Investigational New Drug
ALS: Amyotrophic lateral sclerosis
IRB: Institutional review board
ALSFRS-R: ALS functional rating scale-revised
mITT: Modified intent-to-treat
BP: Blood pressure
MCS: Muscle cramp scale
CD: Cramp diary
MSO: Medical Safety Officer
CGIC: Clinical global impression of changes
NEALS: Northeastern ALS Study Group
CUIMC: Columbia University Irving Medical Center
OHRP: Office for Human Research Protections
DSMB: Data and safety monitoring board
PI: Principal investigator
DCC: Data Coordinating Center
PK: Pharmacokinetic
FDA: Food Drug Administration
PRO: Patient-reported outcomes
GAS: Goal attainment scale
*t*_max_: The time take to reach maximum concentration
GCP: Good Clinic Practice
SAE(s): Serious adverse event (s)
ID: Identification
*t*_1/2_: Elimination half-life
CGIC: Clinical global impression of changes
VAS: Visual analog scale
CUIMC: Columbia University Irving Medical Center
