# Supplementary material for: Randomized double-blind personalized N-of-1 clinical trial to test the safety and potential efficacy of TJ-68 for treating muscle cramps in amyotrophic lateral sclerosis (ALS): study protocol for a TJ-68 trial
Source: Trials. 2023 Jul 10;24:449. doi: 10.1186/s13063-023-07424-8 (PMC10332004; doi:10.1186/s13063-023-07424-8)
Supplement: Supplementary file 1 — Additional file 1: Supplement Table 1. Serious Adverse Events (SAEs). Supplement Table 2. Consideration of Causality of Adverse Events and Expectedness in the Couse of ALS. Supplement Table 3. Dealing with Unanticipated Problems (UPs). Supplement Table 4. Removal of Patients from Study Participation. Supplement Table 5. Participant’s Withdrawal. [file 13063_2023_7424_MOESM1_ESM.docx]

**ONLINE SUPPLEMENT FOR TRIALS**

## **BACKGROUND**

## **TJ-68 Nonclinical Studies**

The pharmacodynamics studies of TJ-68E (TJ-68E: TJ-68’s extract bulk powder without excipients) in animal models identify two effects: peripheral relaxation effect and central analgesic effect. The constituents of peony root, Paeoniflorin and Albiflorin, affect noradrenergic neurons in the descending pain inhibitory pathway of the spinal cord (1). By activating α2 receptors in the spinal cord, they inhibit the release of algogenic substances and provide analgesia. The constituents of Glycyrrhiza, Glycyrrhetic acid, Liquiritin apioside, Liquiritigenin, Isoliquiritin apioside, and Liquiritigenin inhibit intracellular calcium influx and abnormal release of calcium from the sarcoplasmic reticulum (2). They inhibit tetanic contractions exclusively and do not affect physiological twitch contractions of the skeletal muscles (2, 3).

A general pharmacology study suggested that TJ-68E exhibited no major effects on general behavioral, autonomic, neurological, respiratory,cardiovascular, gastrointestinal, or renal functions (4).

For toxicology, neither the Ames test, chromosomal aberration test with mammalian cultured cells, nor the micronucleus test in mice exhibited genotoxicity (5-7). Other toxicology studies also suggested that no-observed-adverse-effect levels (NOAELs) of TJ-68E were more than 10 g/kg in a single dose toxicity test of rats (8); 500 mg/kg/d in a repeat dose toxicity test of rats and dogs (9,10); and 500 mg/kg/d in a reproductive and developmental toxicity test in rats and rabbits (11,12). These NOAELs are much larger than clinical dose levels (2.5-7.5g/day). TJ-68E did not inhibit P-glycoprotein (P-gp) *in vitro* (Caco-2 cells), suggesting that it was unlikely to interact with drugs metabolized by P-gp (13). CYP3A and P-gp were also not inhibited by TJ-68E in mice (14).

##

## **TJ-68 Clinical Studies**

A pharmacokinetic study and several clinical studies were conducted in Japan to confirm the effectiveness of TJ-68 in various disease settings.

In a study of patients with 21 patients with lumbar spinal stenosis, TJ-68 was administered at 7.5 g/day for the first two weeks and then titrated ad libitum to the minimum effective dose by patient. Altogether, 90% of cases achieved 50% cramp reduction. There were very few adverse reactions, including only one case of dizziness (15).

In a randomized double-blind placebo-controlled parallel study of muscle cramps in patients with liver cirrhosis, a total of 101 patients were randomly assigned to receive TJ-68 7.5 g/day (*n* = 52) or placebo 7.5 g/day (*n* = 49) for two weeks. The improvement rate of muscle cramp frequency was significantly superior in the TJ-68 group compared to the placebo-group (*p* = 0.011); 67.3% of patients in the TJ-68 group achieved “Markedly improved’ or ‘Improved’ as compared to 36.7% in the placebo-group. Incidents of adverse reactions were not significantly different between the two groups: 14.3% in the TJ-68 group and 4.9% in the placebo group (*p* = 0.173). The most common adverse reactions included pseudoaldosteronism (n=X, blood-pressure raise, edema, and decrease in serum potassium level) in the TJ-68 group and digestive symptoms in the placebo group; however, no severe adverse reactions were documented (16).

Longer treatment duration was tested in a study of nocturnal leg cramp complicated with diabetic neuropathy. TJ-68 was administered orally at 2.5-7.5 g/day for 4 weeks; 8 out of 12 patients no longer experienced muscle cramps. One patient with more than 2 months of administration had mild hypokalemia. The hypokalemia resolved after discontinuation (17).

A randomized, open-label, two-arm, two-period, crossover pharmacokinetic study using TJ-68 was conducted in twenty Japanese healthy adults (18). TJ-68 was administered as a single dose of 2.5 or 5.0 g/day, with a washout period of at least 7 days to prevent carryover. The absorption of six targeted components (Albiflorin, Albiflorin, Glycycoumarin, Isoliquiritin, Glycyrrhetic acid, and glycyrrhetic acid-3-*O*-monoglucuronide (3MGA)) after the administration of TJ-68 were clarified. The highest plasma concentration was found in Glycyrrhetic acid showing *C*_max_, approximately 200 ng/mL, followed by Paeoniflorin, Paeoniflorin, 3MGA, Isoliquiritin, and Glycycoumarin. Moreover, *t*_max_ of Isoliquiritin, Glycycoumarin, Albiflorin, and Paeoniflorin were found within 3 h after the administration of TJ-68. The constituent notable for the longest *t*_1/2_ was GA showing *t*_1/2_, approximately 10 hours.

As implied by the pharmacokinetic study, the rapid onset of a clinical pharmacological effect was also seen in a study of hemodialysis patients with muscle cramps during hemodialysis sessions. TJ-68 (2.5 g) was administered on an as-needed basis, and muscle cramps and associated pain disappeared in about 5 minutes in 54 out of 61 cases (19).

##

## **Post-Marketing Safety Experience in Japan**

A post-marketing survey was conducted in Japan from 2013 to 2014 by registering 3,330 patients who received TJ-68 with 2,975 patients eventually selected for analysis (20). The overall frequency of adverse drug reactions (ADRs) was 1.1% (33 out of 2,975 patients). The most common adverse events (AEs) were hypokalemia (7 patients [0.2%]) and hypertension, increased blood pressure, edema, face edema, peripheral edema, abdominal discomfort, and nausea (2 or 3 patients [0.1%]). Regarding the onset timing of each ADR, 40.5% of the entire ADRs appeared within 15 days after the start of treatment. On the other hand, hypokalemia tended to appear after treatment was administered for at least 4 weeks. In addition, the ADRs classified into the system organ class of “General disorders and administration site conditions” were recognized throughout the 26-week observation period, irrespective of the length of the treatment period. The incidence rate of ADR was 0.7% (8 out of 1129 patients) in non-elderly patients aged less than 65 years and 1.4% (25 out of 1846 patients) in elderly patients aged at least 65 years. When focusing on the hypokalemia, the most frequent ADR was seen in 7 of 33 patients (all of the 7 patients were elderly patients aged at least 65 years). Two ADRs in one patient (aged 80 years) were judged as serious (hypokalemia and hypertension, respectively). The remaining 35 ADRs in 32 patients were judged as not serious. There was no statistically significant correlation between the incidence rate of ADR and patient variables, such as gender, age, BMI, allergy history, liver dysfunction, and renal dysfunction.

In a retrospective cohort study investigating the occurrence of hypokalemia in patients treated with Yokukansan, another licorice-containing Kampo preparation, the following risk factors were found; age, duration of treatment,: dosage, co-administration of potassium-lowering drug, and hypoalbuminemia (21).

As described previously, TJ-68 has been widely used (e.g., about 2,300,000 estimated annual patients in the year 2019) in Japan since its regulatory approval in 1986. In spontaneous safety reporting, 566 serious and 1055 non-serious ADRs were reported between 1994 and 2020. The most frequent ADRs among the serious ADRs involved pseudoaldosteronism, hypokalemia, rhabdomyolysis, myopathy, and other cardiac disorders due to hypokalemia. Other minor ADRs involved interstitial lung disease and hepatic dysfunction. Most adverse event reports were among older populations, which we believe is the age group most likely to use the preparation.

Hypokalemia and pseudoaldosteronism are presumed to be ADRs caused by Glycyrrhiza/GL, but the relation between the other ADRs and patient variables or risk factors are unknown. Characteristic ADR derived from peony root was not found.

**COLLECTION AND EVALUATION OF AE/SAE AND REPORTING**

**Recording and Reporting AEs and SAEs**

**Adver Event (AE)**

As defined by the International Conference on Harmonization, an AE is any untoward medical occurrence in a participant following the administration of an investigational product. This is not necessarily indicative of a causal relationship between the investigational product and AE. An AE can therefore be any unfavorable and unintended sign (including an abnormal laboratory finding), symptom, or disease (new or exacerbated) temporally associated with the use of the investigational product.

## **Serious Adverse Events (SAEs)**

A serious adverse event (SAE) is any AE in the following situations, summarized in Table 1.

## **Collection of AEs/SAEs**

During each scheduled visit, ask about potential AEs or SAEs using the following standard questions:

1. Have you had any medical problems since your last visit?
2. Have you taken any new medications since your last visit?

## **Recording and Reporting AEs**

Events occurring between the Screening Visit and just prior to the first dose of the investigational product on Day 1 should be recorded in the Medical History eCRF unless the event is related to a protocol-mandated procedure. If the event is deemed related to a protocol-mandated procedure (e.g., hematoma at the puncture site) and deemed clinically significant by the Investigator, the event should be reported as an AE or an SAE (if applicable).

AEs will be documented from the first administration of the investigational product through the Follow-Up Visit. The Principal Investigator at each study site (CU and Mayo Clinic) will review all documentation (e.g., hospital progress notes, laboratory and diagnostic reports) relevant to the event. A diagnosis will then be determined based on signs, symptoms, and/or other clinical information. The diagnosis and not the individual signs/symptoms should be reported as the AE term.

ALS progression and signs/symptoms that are expected as part of ALS progression will not be collected as AEs unless they meet seriousness criteria and should be recorded as SAEs.

## **Recording and Reporting SAEs**

If an AE meets any of the seriousness criteria, it must be reported using the SAE report form **within 24 hours of the site’s knowledge to the Columbia University Study Center**. At a minimum, the following information should be included:

• Patient number

• Event term, including an onset date and stop date, if applicable, and a brief description

• Seriousness criterion/criteria

• Causality assessment in relation to the investigational product

If all information regarding the SAE is not initially available, the sites (CU and Mayo Clinic study sites) should still report the SAE within 24 hours of awareness/discovery. Additional information should be reported when it becomes available and no later than 24 hours after receipt of such information.

The Sponsor will be responsible for notifying the Food and Drug Administration (FDA) of any unexpected fatal or life-threatening suspected adverse reaction as soon as possible, but in no case later than 7 calendar days after the Sponsor's initial receipt of the information. In addition, the Sponsor must notify FDA and all participating investigators in an Investigational New Drug (IND) safety report of potential serious risks, from clinical trials or any other source, as soon as possible, but in no case later than 15 calendar days after the sponsor determines that the information qualifies for reporting according to 21 CFR 312.32.

## **Evaluating AEs and SAEs**

### Assessment of Severity

The Investigator should assess the severity of each AE/SAE. The severity of AEs/SAEs will be assessed by assigning a Grade of 1, 2, 3, 4 or 5 according to the National Cancer Institute Common Terminology Criteria for Adverse Events (CTCAE), version 6.0. (<https://ctep.cancer.gov/protocoldevelopment/electronic_applications/ctc.htm#ctc_60> )

The Investigator will report UPs to the Data Coordinating Center (DCC)/Sponsor using research electronic data capture system (REDCap). The UP report will include the following information (see **Table 3**). To satisfy the requirement for prompt reporting, UPs will be reported using the following timeline outlined in **Table 3**.

##

**References**

1.      Lee KK, Omiya Y, Yuzurihara M, Kase Y, Kobayashi H. Antinociceptive effect of paeoniflorin via spinal alpha(2)-adrenoceptor activation in diabetic mice. Eur J Pain 2011 Nov;15(10):1035-1039. Epub 20110518.

2.      Lee KK, Omiya Y, Yuzurihara M, Kase Y, Kobayashi H. Antispasmodic effect of shakuyakukanzoto extract on experimental muscle cramps in vivo: Role of the active constituents of Glycyrrhizae radix. J Ethnopharmacol 2013 9 January;145(1):286-293.

3.      Kaifuchi N, Omiya Y, Kushida H, Fukutake M, Nishimura H, Kase Y. Effects of shakuyakukanzoto and its absorbed components on twitch contractions induced by physiological Ca2+ release in rat skeletal muscle. J Nat Med 2015 Jul;69(3):287-295. Epub 20150318.

4.      Takeda S, Goto K, Ishige A, Wei JW, Ruo TI, Cheng FC, Aburada M. General pharmacological properties of shakuyaku-kanzo-to. Ōyō yakuri/Pharmacometrics 2003;64(1/2):23-31.

5.      Kuboniwa H. Bacterial Reverse Mutation Study of TSUMURA Shakuyakukanzoto (TJ-68). Tsumura Research Laboratories, Tsumura & Co.; Ibaraki, Japan: Laboratory Report Number 1992-0616/M-91-002; 1993 February 22

6.      Ohta K. Chromosomal Aberration Study of TSUMURA Shakuyakukanzoto (TJ-68) in CHL/IU. Kagoshima Main Branch Drug Safety Research Laboratories, Shin Nippon Biomedical Laboratories, LTD.; Kagoshima, Japan: Laboratory Report Number 2012-0346/SBL76-43. 1993 March 23

7.      Nagasawa K. Micronucleus Test of TSUMURA Shakuyakukanzoto (TJ-68) with Mice Bone Marrow Cells. Gifu Laboratories. Nihon Bioresearch Inc.; Gifu, Japan: Laboratory Report Number 2012-0345/9521; 1993 March 25

8.      Papagiannis C. Acute Oral Toxicity Study of TJ-68 in Rats - Limit Test. International Research and Development Corporation.; Michigan, U.S.A.: Laboratory Report Number 2012-0337/661-005; 1993 November 16

9.      Spicer B. 13- Week Oral Toxicity Study of TJ-68 in Rats with a One Month Recovery Period. International Research and Development Corporation.; Michigan, U.S.A.: Laboratory Report Number 2012-0338/661-002; 1994 March 25

10.      Namiki M. Three-Month Repeated Oral Dose Toxicity Study of TSUMURA Shakuyakukanzoto with 1-Month Recovery Study in Dogs. Hokkaido Laboratories, Safety Research Institute for Chemical Compounds Co., Ltd.; Hokkaido, Japan: Laboratory Report Number 2017-0217/SR16292; 2017 September 29

11.      Fujii S. Study for Effects of TSUMURA Shakuyakukanzoto on Embryo-Fetal Development in Rats. Hokkaido Laboratories, Safety Research Institute for Chemical Compounds Co., Ltd.; Hokkaido, Japan: Laboratory Report Number 2019-0195/SR18099; 2019 March 20

12.      Yabe K. Study for Effects of TSUMURA Shakuyakukanzoto on Embryo Fetal Development in Rabbits. Hokkaido Laboratories, Safety Research Institute for Chemical Compounds Co., Ltd.; Hokkaido, Japan: Laboratory Report Number 2019-0194/SR18097; 2019 March 20

13.      Matsumoto T, Kaifuchi N, Mizuhara Y, Warabi E, Watanabe J. Use of a Caco-2 permeability assay to evaluate the effects of several Kampo medicines on the drug transporter P-glycoprotein. J Nat Med 2018 Sep;72(4):897-904. Epub 20180524.

14.      Matsumoto S. Effects of Shakuyakukanzoto Extract (TJ-68E) Cyp3a and P-glycoprotein *In Vivo*Mouse. LSI medience corporation.; Tokyo, Japan: Laboratory Report Number 2017-0125/B161145; 2017 July 24

15.      Takao Y, Takaoka Y, Sugano A, Sato H, Motoyama Y, Ohta M, Nishimoto T, Mizobuchi S. Shakuyaku-kanzo-to (Shao-Yao-Gan-Cao-Tang) as treatment of painful muscle cramps in patients with lumbar spinal stenosis and its minimum effective dose. Kobe J Med Sci 2015;61(5):E132-E137.

16.      Kumada T, Kumada H, Yoshiba M, Nakano S, Suzuki H, Tango T. Effects of Shakuyaku-kanzo-to (Tsumura TJ-68) on muscle cramps accompanying cirrhosis in a placebo-controlled double-blind parallel study. J Clin Ther Med 1999;15:499-523.

17.      Miura Y. Effects of Shakuyaku-kanzo-to on painful nocturnal leg cramp caused by diabetic neuropathy. Jpn J Orient Med 1999;49(5):865-869.

18.      Sadakane C, Watanabe J, Fukutake M, Nisimura H, Maemura K, Kase Y, Kono T. Pharmacokinetic Profiles of Active Components After Oral Administration of a Kampo Medicine, Shakuyakukanzoto, to Healthy Adult Japanese Volunteers. J Pharm Sci 2015 Nov;104(11):3952-3959. Epub 2015 Jul 24.

19.      Hyodo T, Taira T, Takemura T, Yamamoto S, Tsuchida M, Yoshida K, Uchida T, Sakai T, Hidai H, Baba S. Immediate effect of Shakuyaku-kanzo-to on muscle cramp in hemodialysis patients. Nephron Clin Pract 2006;104(1):c28-32. Epub 2006 May 09.

20.      Maki A., Hisada T, Katori Y. Adverse Drug Reaction Frequency Investigation of TSUMURA Shakuyakukanzoto Extract Granules for Ethical Use  . Diagnosis and treatment 2016;104(7):947-958.

21.      Shimada S, Arai T, Tamaoka A, Homma M. Liquorice-induced hypokalaemia in patients treated with Yokukansan preparations: identification of the risk factors in a retrospective cohort study. BMJ Open 2017 Jun 15;7(6):e014218-2016-014218. Epub 20170615.

**Supplement Table 1.**

| **Serious Adverse Events (SAEs)** | |
| --- | --- |
| 1. | **Results in Death** |
|  | Death is selected as a seriousness criterion ONLY when the event is the cause of death. Death is an outcome and the event which led to the death should be the reported event term. |
| 2. | **Life-Threatening Events** |
|  | The term 'life-threatening' in the definition of 'serious' refers to an event in which the patient was at risk of death at the time of the event. It does not refer to an event that hypothetically might have caused death if it were more severe, prolonged, or untreated. |
| 3. | **Requiring Hospitalization or Prolongation of Existing hospitalization** |
|  | Hospitalization signifies that the subject has been admitted to the hospital as an in-patient for any length of time. Emergency room treatment does not qualify for this category but may be appropriately included (see below). If a complication prolongs the hospitalization or fulfills any other seriousness criterion or criteria, the complication will be considered an additional SAE. When in doubt as to whether ‘hospitalization’ occurred, we will consult the Medical Safety Officer.  Hospitalization should not be considered an AE term in itself. It will be considered an outcome of an AE. For example, hospitalization for an elective treatment of a pre-existing condition that did not worsen after the first dose of the investigational product will not be considered an AE. |
| 4. | **Results in Disability, Incapacity, or Both** |
|  | The term disability means a substantial or permanent disruption of a person’s ability to conduct normal life functions. This definition is not intended to include experiences of relatively minor medical significance, such as uncomplicated headache, nausea, vomiting, diarrhea, influenza, and accidental trauma (e.g., sprained ankle) that may temporarily interfere with or prevent everyday life functions but do not constitute a substantial or permanent disruption. |
| 5. | **An Important Medical Event** |
|  | Important medical events that may not be immediately life-threatening or result in death or hospitalization but may jeopardize the patient or may require medical or surgical intervention to prevent one of the other outcomes listed in the above definition may be considered serious. |

The definition of SAE is similar to any protocol. It is adopted from various previous study protocols.

**Supplement Table Supplement 2. Consideration of Causality of Adverse Events and Expectedness in the Couse of ALS**

| **Assessment of Causality** | | |
| --- | --- | --- |
|  | • Temporal association between the administration of the investigational product and the event  • Cessation of the AE following discontinuation of dosing  • Recurrence of the AE with reintroduction of study drug, if performed  • Similarity to known class effects  • Alternative causes, such as known effects of concomitant medications  − Pre-existing risk factors  − Concurrent illnesses | |
| **Assessment of Expectedness: Based on Medical Dictionary for Regulatory Activities (MedDRA)** | | |
|  | Dysarthria  Dysphagia  Dyspnea  Gait disturbance  Involuntary muscle contractions  Muscle spasms | Muscle spasticity  Muscle weakness  Muscle stiffness  Pneumonia aspiration  Respiratory failure  Weight loss |

**Supplement Table 3.** **Dealing with** **Unanticipated Problems (UPs)**

| **Unanticipated Problems (UPs)** | |
| --- | --- |
| 1. **Unanticipated problems (PUs) involving risks to participants meeting all of the following criteria** | |
|  | Unexpected in terms of nature, severity, or frequency given (a) the research procedures that are described in the protocol-related documents, such as the IRB-approved research protocol and informed consent document; and (b) the characteristics of the participant population being studied;  Related or possibly related to participation in the research (“possibly related” means there is a reasonable possibility that the incident, experience, or outcome may have been caused by the procedures involved in the research); and  Suggests that the research places participants or others at a greater risk of harm (including physical, psychological, economic, or social harm) than was previously known or recognized. |
| 1. **The UP reporting to DCC (into REDcap) will include the following information** | |
|  | - Protocol identifying information: protocol title and number, PI’s name, and the IRB project number; - A detailed description of the event, incident, experience, or outcome; - An explanation of the basis for determining that the event, incident, experience, or outcome represents an UP; - A description of any changes to the protocol or other corrective actions that have been taken or are proposed in response to the UP. |
| 1. **The UPs will be reported using the following timeline** | |
|  | - Investigator will report UPs to the DCC/Sponsor within 24 hours of the Investigator becoming aware of the event. - Investigator should also report UPs to their local IRB as required by their institution’s written reporting procedures. - Lead PI should report all UPs promptly to the IRB of Record, but no later than one week following - the occurrence of the UP or the PI’s acquiring knowledge of the UP for the events that occurred at the lead site; and - the determination that the occurrence meets the UP criteria for the events that occurred at other study sites. |

## **Supplement Table 4.** **Removal of Patients from Study Participation**

| **Patients may discontinue the study drug or withdraw from the study due to the following:** |
| --- |
| - A change in compliance with inclusion/exclusion criteria that is clinically relevant and/or affects patient safety, and/or study assessments/objectives, etc. - Occurrence of intolerable AEs - Changes in vital signs, ECGs, or clinical laboratory results that, in the opinion of the Investigator, pose a significant health risk - Intake of non-permitted concomitant medication that might affect patient safety or study assessments/objectives, etc. - Severe hypokalemia during a routine laboratory study, requiring in-hospital treatment - When impending hypokalemia is detected (potassium level 3.1-3.4 mEq/L or lower, start KCL 20 mEq BID, repeat in 3 days, if remains 3.4 mEq/L or lower, stop the drug - Having a potassium level of 3.0 mEq/L or lower is immediate stop of the drug (simultaneously start KCl 20 mEq TID and repeat potassium in 3 days) - Having DBP higher than 100 mmHg or SBP higher than 160 mmHg - eGFR calculated using the CKD-EPI cystatin equation (54) shows a >25% decline compared to baseline (defined as Day 1 or Screening if Day 1 value is not available) as long as the decline is not related to dehydration or a superimposed reversible process such as infection or nausea - Changes in LFTs as outlined in Appendix C - Receiving any form of stem cell therapy for ALS either as part of a clinical trial or outside of a clinical trial - Having a diaphragmatic pacing system implanted either as part of a clinical trial or outside of a clinical trial - Initiating edaravone after screening - Initiating or increasing riluzole after screening - Initiating gene therapy for ALS either as part of a clinical trial or outside of a clinical trial after screening - Unusual worsening of ALS or muscle cramps that, in the opinion of the Investigator, pose a significant health risk |

**Supplement Table 5**. **Participant’s Withdrawal**

| **Main Reasons for Participant’s Withdrawal From the Study** | |
| --- | --- |
|  | 1. Patient Death 2. AE: One or more clinical or laboratory events which, in the medical judgment of the Investigator, are grounds for discontinuation even if the event does not appear to be related to study medication. The patient may withdraw because of an AE even if the Investigator does not feel that the event is grounds for discontinuation. 3. Protocol Violation: The patient’s findings or conduct failed to meet the protocol entry criteria or failed to adhere to the protocol requirements. 4. Patient Withdrawal of Consent: Patient desires to withdraw from further participation in the study and provides a reason. 5. Administrative/Other: Any cause of premature termination from the study other than the above, such as illness of investigator, loss of study drug, or termination of study by the Sponsor. |
